# Salinity Effect on Germination and Further Development of Parasitic *Cuscuta* spp. and Related Non-Parasitic Vines

**DOI:** 10.3390/plants10030438

**Published:** 2021-02-25

**Authors:** Lyuben Zagorchev, Alexandra Atanasova, Kalina Pachedjieva, Anita Tosheva, Junmin Li, Denitsa Teofanova

**Affiliations:** 1 Zhejiang Provincial Key Laboratory of Plant Evolutionary Ecology and Conservation, Taizhou University, Taizhou 318000, China; lijmtzc@126.com; 2 Faculty of Biology, Sofia University “St. Kliment Ohridski”, 8 Dragan Tsankov blvd., 1164 Sofia, Bulgaria; atanassova.y@gmail.com (A.A.); kalina.pachedjieva@biofac.uni-sofia.bg (K.P.); atosheva@biofac.uni-sofia.bg (A.T.); teofanova@biofac.uni-sofia.bg (D.T.)

**Keywords:** abiotic stress, dormancy, parasitic plants, weeds

## Abstract

Plants are continuously subjected to the unfavorable impact of abiotic stress factors, of which soil salinity is among the most adverse. Although away from direct soil contact throughout most of their lifecycle, stem parasitic plants of the genus *Cuscuta*, family Convolvulaceae are also affected by salinity. The present study aimed to assess salt stress impact on germination and early establishment of three *Cuscuta* species, in comparison to related nonparasitic vines of the same family. It was found, that *Cuscuta* spp. are highly sensitive to NaCl concentration within the range of 200 mM. Germination was delayed in time and reduced by nearly 70%, accompanied by decrease in further seedling growth, ability to infect host plants and growth rate of established parasites. The nonparasitic vines showed similar sensitivity to salinity at germination level, but appeared to adapt better after the stress factor was removed. However, the negative effect of salinity did not fully prevent some of the *Cuscuta* species from infecting hosts, probably a beneficial characteristic at a species level, allowing the parasite to successfully thrive under the scarce host availability under saline conditions.

## 1. Introduction

Salinity stress is among the most damaging environmental factors, affecting plant growth and development worldwide, also leading to increasing crop yield losses [1]. Although some plant species, called halophytes, are naturally adapted to saline conditions and could sustain extreme salt concentrations [2], the predominant flora, respectively most of the crop plants are glycophytes, e.g., salt-sensitive. As such, the question of improvement of crop salt tolerance [1,3] has been a major topic in contemporary scientific literature for decades.

Salts affect plants’ lifecycles at every possible stage, from breaking dormancy and early germination events through vegetation to flowering and seed yield [4]. First, soil salinity leads to decreased osmotic potential, preventing or impeding imbibition and seed germination. Sodium chloride concentration as low as 50 mM could lead to over 75% decrease of germination percentage of some species (*Arabidopsis thaliana* (L.) Heynh. ecotype Col, [4]). Certain halophytes, although also challenged by extreme salinity, may retain germination ability above 10% at concentrations as high as 1 M or greater [5]. Seed germination ability under salinity of a particular species may vary significantly, based on genotypic differences [6], seed heteromorphism [7] and may be altered by seed priming with natural or synthetic compounds [8]. The complex response of seeds to salt stress during germination is generally managed by gibberellic acid [9], abscisic acid [10] or ethylene and nitric oxide [11] triggered pathways. Further plant development is negatively affected by salinity through the extensively studied mechanisms of osmotic stress and ion toxicity [12], combated by ion exclusion/sequestration [13], compatible solute accumulation [14] and antioxidant response [15].

Abiotic stress factors also lead to substantial changes of plant communities, affecting biodiversity and organismal interactions. Particularly salinity affects soil microbial biodiversity [16] and shapes plant communities by benefiting slow growing halophytes over glycophytes [17]. Next, the arbuscular mycorrhizal symbiosis is also largely affected [18], as well as plant susceptibility to various diseases, which may either increase [19] or decrease [20].

A special case here represents the interactions between parasitic plants and their respective hosts. Although few of them are considered halophytes, they are also known to inhabit salinized lands and parasitized on halophytic hosts [21]. Some, like *Cuscuta salina* Engelm. significantly shape plant species biodiversity in salt marshes [22] and coastal wetlands [23] by preferentially infecting and thus restricting certain species and avoiding others. Salinity might change host preferences of the parasite. It is experienced by the parasite through metabolic and physiological changes in the host [24] and simultaneously the parasite may impair the proper response to salinity of its host [25].

Of approximately 4000 known parasitic flowering plants and 200 in the family Convolvulaceae, *Cuscuta campestris* Yunck. is among the most prominent. It is a North American species, currently with worldwide distribution [26], highly invasive and challenging for crop production in numerous countries [27]. It is a stem holoparasitic plant with generalist preferences—infecting numerous host plant species simultaneously. Although not considered a typical halophyte specialist like its relative *C. salina*, in 2018 it was found on the Bulgarian sea coast, parasitizing *Centaurea arenaria* Bieb. & Willd. on a sand dune (NG 67, 42°13′50″ N 27°46′35″ E, herbarium SO 107977 of Sofia University). Its high ecological plasticity raises concerns that it might spread beyond optimal conditions and damage fragile ecosystems like coastal lands and pose additional threat to the emerging agriculture in salinized areas. Its parasitic potential under salt stress conditions, however, is not extensively studied.

The aim of the present study was to evaluate the effect of salinity on germination and parasitic development of *C. campestris* in comparison to two other *Cuscuta* species, the widely distributed Asian species *Cuscuta chinensis* Lam. and *Cuscuta japonica* Choisy., as well as two nonparasitic members of Convolvulaceae family *Ipomoea tricolor* Cav., *Convolvulus arvensis* L. and *Calystegia sepium* (L.) R. Br. The choice of *C. chinensis* was driven by the phenotypical similarity with *C. campestris* [28], while *C. japonica* was chosen due to its contrasting phenotype to *C. campestris* and its recent status of noxious weed in North America, suggesting its invasive potential [29]. Of the nonparasitic species, *I. tricolor* is a popular ornamental plant, while *Convolvulus arvensis* and *Calystegia sepium* are widely distributed weed species [30]. Overall, numerous species within the family are fast-growing vines with weed potential.

## 2. Results

### 2.1. Effect of Salinity on Germination and Seedling Growth

**Figure 1:**
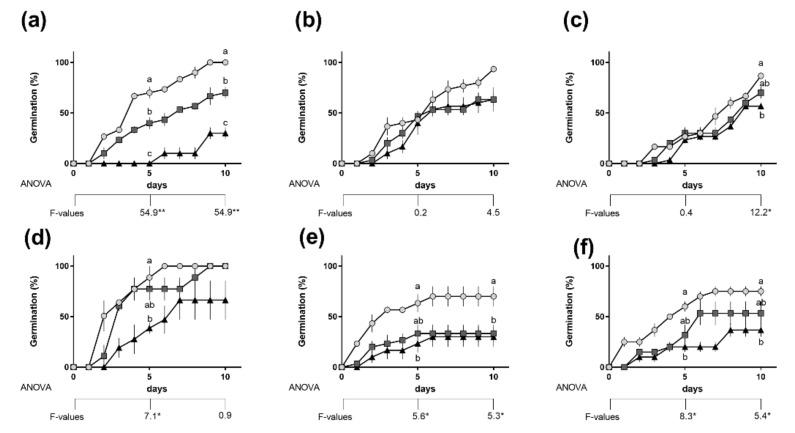
Germination rate of *Cuscuta campestris* (**a**), *Cuscuta chinensis* (**b**), *Cuscuta japonica* (**c**), *Ipomoea tricolor* (**d**), *Convolvulus arvensis* (**e**) and *Calystegia sepium* (**f**) at 0 (circles), 100 mM (squares) and 200 mM (triangles) NaCl. Different letters indicate statistical significance at *p* ≤ 0.05 (Tukey’s post hoc test), one-way ANOVA analysis (* *p* ≤ 0.05; ** *p* ≤ 0.01). n = 3. Levene’s homogeneity test: *p* > 0.05. Presented values are mean ± SEM.

Decrease in germination rate was different among the studied species. The most significant effect of salinity was observed in *C. campestris* where decrease in germination of nearly 70% and delay up to the 5th day at 200 mM NaCl were detected (Figure 1a). The inhibitory effect was nonsignificant (*C. chinensis*, Figure 1b) or less significant (*C. japonica*, Figure 1c) in the other two parasitic species, where 100 and 200 mM NaCl had similar effect and caused reduction in germination by approximately 20%. Among the nonparasitic plants, both salt concentrations caused similar effect and around 40% inhibition in *C. arvensis* (Figure 1e), while in *I. tricolor* only a short delay in germination was observed at 100 mM NaCl with an overall similar germination rate (Figure 1d). In *C. sepium* the response was similar to *C. campestris*, although less pronounced (Figure 1f)—20% lower germination at 100 mM and 40% lower at 200 mM NaCl as compared to controls. 

The significance of effect of species and salinity on final germination percentage (10th day) was further tested (Table 1). While the effect of salinity was significant at *p* ≤ 0.01, no significant effect was found for the species or the combination of species and salinity.

**Figure 2:**
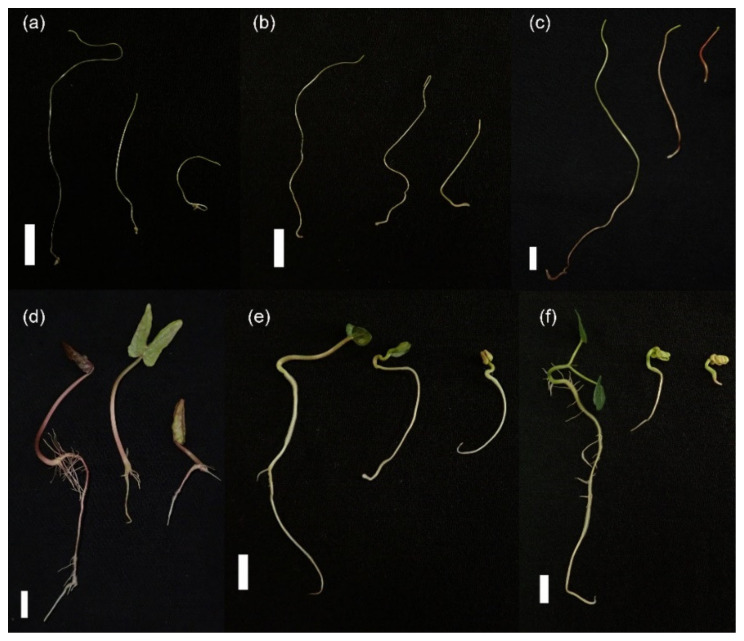
Seven days old seedlings of *Cuscuta campestris* (**a**), *Cuscuta chinensis* (**b**), *Cuscuta japonica* (**c**), *Ipomoea tricolor* (**d**), *Convolvulus arvensis* (**e**) and *Calystegia sepium* (**f**) at 0, 100 mM and 200 mM NaCl (from left to right). Scale bar = 1 cm.

**Figure 3:**
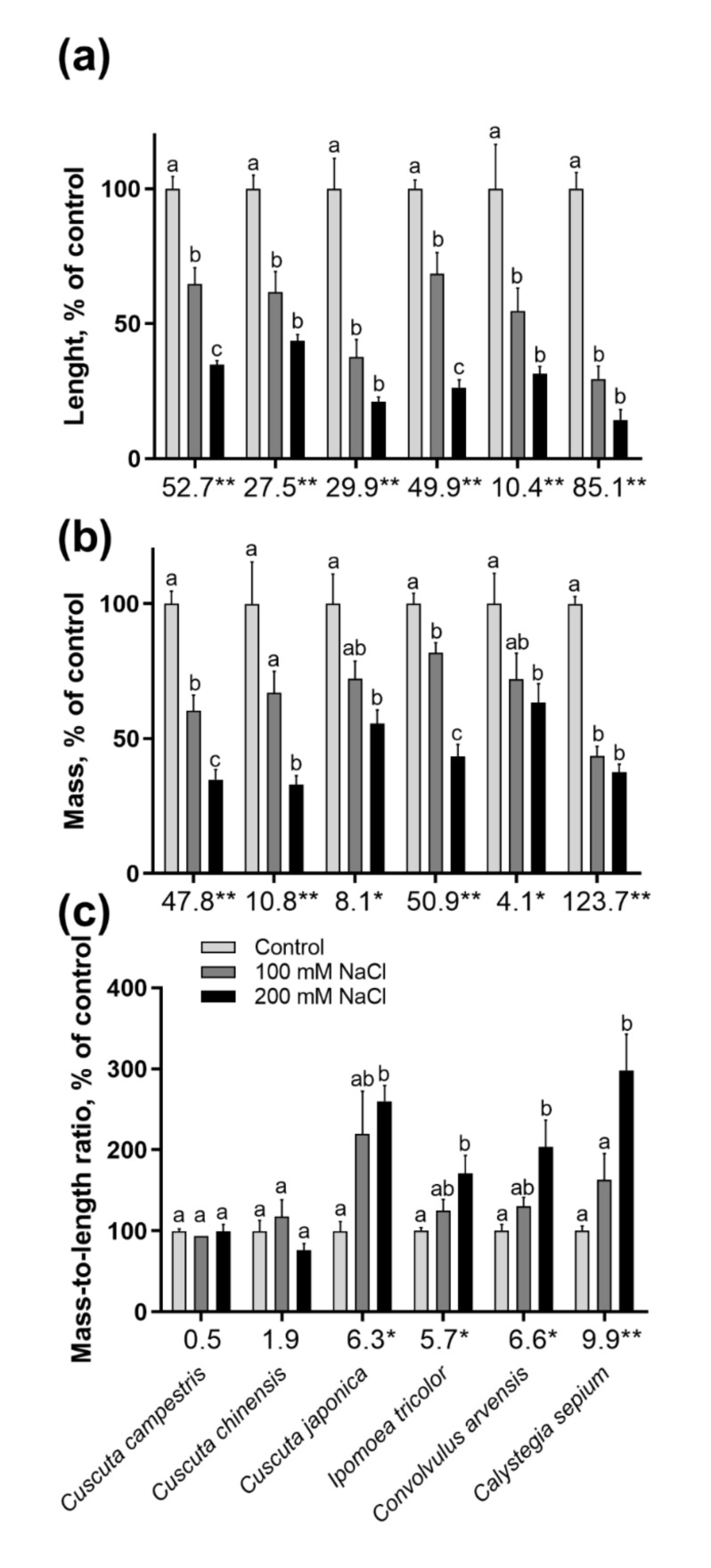
Seedlings growth at 0, 100 mM and 200 mM NaCl. Growth was calculated as cm day-1 (**a**), mg day-1 (**b**) and mass-to-length (mg cm^−1^) ratio (**c**) and presented as percent of control. Different letters indicate statistical significance at *p* ≤ 0.05 (Tukey’s post hoc test), one-way ANOVA analysis (* *p* ≤ 0.05; ** *p* ≤ 0.01). n = 6. Levene’s homogeneity test: *p* > 0.05. Presented values are mean ± SEM.

Further growth of seedlings was monitored at the 7th day after germination, revealing a similar inhibiting effect (Figure 2). Salinity also affected negatively lateral roots growth in the nonparasitic species (Figure 2d–f). In *Cuscuta* spp. the root is highly rudimentary [31] and no visible differences were found or expected with salinity treatment. Seedling length (Figure 3a) and fresh mass (Figure 3b) were decreased by over 50% at 200 mM NaCl in all species. *Cuscuta japonica* and *C. sepium* showed the most pronounced inhibition of growth by salinity, even at 100 mM NaCl, although these are not the most affected species in terms of germination (Figure 1).

Opposite to seedlings’ length and mass, the mass-to-length ratio increase with salinity concentration in all nonparasitic species and in *C. japonica* (Figure 3c), while differences in *C. campestris* and *C. chinensis* were nonsignificant.

### 2.2. Hydrolytic Enzymes in Seeds

**Figure 4:**
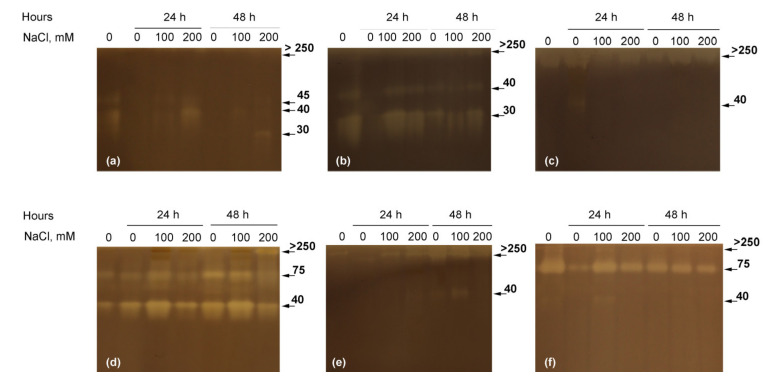
Amylase isoforms in germinating seeds of *Cuscuta campestris* (**a**), *Cuscuta chinensis* (**b**), *Cuscuta japonica* (**c**), *Ipomoea tricolor* (**d**), *Convolvulus arvensis* (**e**) and *Calystegia sepium* (**f**) at 0, 100 mM and 200 mM NaCl at 24th and 48th hour. Each isoform is indicated with its calculated molecular weight in kDa.

Two major classes of hydrolytic enzymes with a possible role in seeds’ storage compounds were studied at the 24th and 48th hour of germination. A total of 5 differing in molecular weight bands of amylolytic activity were detected in all studied species (Figure 4). The larger was distributed on the top of the separating gel and was therefore denoted as larger than 250 kDa (the highest protein standard). We do not exclude the possibility that it was composed of several α-amylase isoforms. In *Cuscuta* spp. most of the amylolytic bands were more pronounced at salt treatments and during the 24th hour (Figure 4a,b) with the exception of *C. japonica*, where salt treatment seemed to inhibit amylolytic activity (Figure 4c). In the nonparasitic species, and especially in *I. tricolor* and *C. sepium* (Figure 4d,f) the activity was much more uniform, but also with increase at 100 mM NaCl in comparison to controls and 200 mM NaCl-germinating seeds.

**Figure 5:**
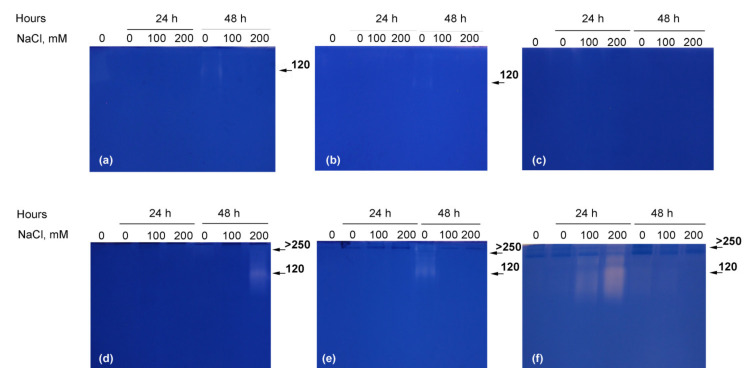
Protease isoforms in germinating seeds of *Cuscuta campestris* (**a**), *Cuscuta chinensis* (**b**), *Cuscuta japonica* (**c**), *Ipomoea tricolor* (**d**), *Convolvulus arvensis* (**e**) and *Calystegia sepium* (**f**) at 0, 100 mM and 200 mM NaCl at 24th and 48th hour. Each isoform is indicated with its calculated molecular weight in kDa.

Unlike amylases, the proteolytic activity in germinating seeds was significantly lower and fewer bands were detected (Figure 5). A single band was detected at the 48th hour in control seeds in *C. campestris* and *C. chinensis* (Figure 5a,b), while no protease at all was detected in *C. japonica* (Figure 5c). A similar pattern was observed in *Convolvulus arvensis* (Figure 5e), while in *I. tricolor* activity was detected in 200 mM NaCl-germinating seeds (Figure 5d). In *Calystegia sepium*, however, proteases were activated by salinity and visible at the 24th hour (Figure 5f).

### 2.3. Effect of Salinity on Subsequent Growth

The germinated seedlings of both parasitic and nonparasitic plants were further transferred to soil (with *A. thaliana*, serving as host for *Cuscuta*) and monitored for growth and survival rates. Salinity priming had a certain negative effect on *Cuscuta*’s ability to infect host plants and the time periods for infection and secondary stem formation. Pregermination under saline conditions reflected in longer time for attachment to host (a delay with 3–4 days) and longer time for secondary stem formation (a delay with 2–3 days). Simultaneously, about 15% of the *C. campestris* seedlings, germinated under 100 mM NaCl and nearly 50% of those under 200 mM NaCl failed to successfully infect host plants. The effect at 100 mM NaCl was similar in *C. chinensis* and *C. japonica*, while at 200 mM NaCl both species failed to attach to the host. This, however, proved to be the critical developmental step for the parasite, as further development, defined as number of plants able to form secondary stem, was not impeded by salinity pretreatment. In the nonparasitic species all seedlings, transferred to soil developed and albeit at a slower rate than controls, grew successfully.

**Figure 6:**
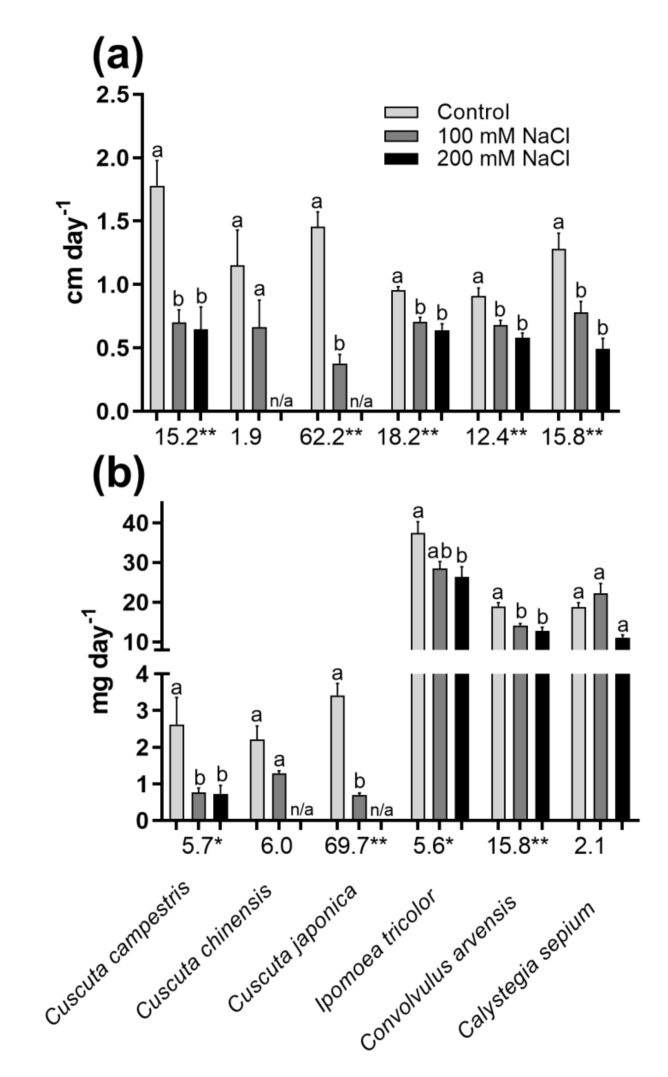
Growth rate in cm day-1 (**a**) and mg day-1 (**b**) of seedlings, germinated under different NaCl concentrations on host Arabidopsis thaliana (Cuscuta spp.) or soil (other species). Data represent mean ± SE. Different letters represent statistical significance at *p* ≤ 0.05, Tukey’s post hoc test. One-way ANOVA F-values are given below, * *p* ≤ 0.05, ** *p* ≤ 0.01. Levene’s homogeneity test: *p* > 0.05.

Growth rate after transfer to saline soil or secondary stem formation on nonstressed hosts was calculated as either cm day^−1^ or mg day^−1^ (Figure 6). It was assumed that potential hosts encountered by the parasite would be adapted to salinity and therefore not stressed, while the nonparasitic relative would be further challenged by soil salinity. A similar inhibition of growth rate at 100 and 200 mM NaCl was observed in all species. While the decrease in the nonparasitic species was within 20–30%, the effect of germination under saline conditions in *Cuscuta* was higher, leading to over 70% decrease in growth rate compared to controls (Figure 6).

## 3. Discussion

### 3.1. Seed Germination Differences Are Related to Parasitic Lifestyle

The comparative analysis of parasitic and nonparasitic members of Convolvulaceae seed germination showed slower germination in the parasitic plants, further delayed by salinity (Figure 1), which is in conformity with the existing literature. The strategy of nonparasitic vines involves fast initial growth and climbing over other plants to outcompete surrounding species [32]. The relatively big seeds of these species germinated as fast as possible (Figure 1d–f), mobilizing starch by activation of amylases (Figure 4). In contrast, even when physical dormancy was artificially broken (through scarification), germination in *Cuscuta* spp. was slower.

The ecological strategy of the parasitic species involves strong physical and possibly physiological dormancy [33], ensuring long seed persistence in soil and continuous germination over several years. The slower germination (Figure 1a–c) would allow emergence when potential host species are already established, considering that although a limited photosynthetic capacity [34,35] may support initial growth, it is not sufficient and along with impaired ability to absorb minerals from the soil due to gene loss [36] do not ensure survival in host absence. Therefore, the initial seedling growth in *Cuscuta* is directed toward immediate attachment to a potential host. Typically, the highest infectious potential of the seedlings is observed in the first 5–7 days, when the events of host attachment and further haustoria and secondary stem formation were fast to immediate. Under control conditions, the observed germination rate of 100% (Figure 1a–c) is also in conformity with previous reports [33] and evidence, supporting that unlike other parasitic plants [37], breaking of physical dormancy in *Cuscuta* spp. is sufficient to induce germination and no host-released germination stimulants are required.

The involvement of storage compounds’ mobilization in germinating seeds was shown to be similar in all species, activated toward the 48th hour with prevalence of amylase activity (Figure 4). Dependence on amylolytic activity was further increased by salinity and especially relevant for the parasitic plant, where the highest activity was observed under salinity, already activated in the first 24 h under 100 mM NaCl, with a delay toward the 48th hour under 200 mM NaCl (Figure 4). This pattern suggests an increased role of stored starch in *Cuscuta* germination under suboptimal conditions, but also fast depletion of the existing resources, in conformity with the overall tendency of parasitic plants to have small seeds, poor in storage compounds [38]. This allows the production of large amount of seeds by a single plant, a general strategy for longer persistence in parasitic plants [39], but apparently is also a negative factor under stress conditions. Proteases (Figure 5) were shown to be less involved in seed germination, especially in the parasitic species.

### 3.2. Salinity Negatively Affects but Does Not Obliterate *Cuscuta campestris* Parasitic Ability

Apparently, the parasitic *Cuscuta* were shown to be much more sensitive to salinity than their nonparasitic relatives. Not only were germination rate (Figure 1) and growth in host absence (Figure 2 and Figure 3) negatively affected, but also the further ability to infect potential hosts and growth rates after establishment (Figure 6). This was especially true for *C. campestris*, a widely distributed invasive species, while the other two *Cuscuta* species proved to be less sensitive to salinity in terms of germination (Figure 1), but also less successful at the later developmental stages. Generally, *Cuscuta* spp. are known to germinate under wide amplitudes of environmental conditions [33,40]. Such plasticity is largely related to the possibility that if a suitable host plant is available, then the further growth of the parasite is ensured. Some authors reported insensibility of *Cuscuta* seedlings to stress-responsive ABA signaling [41], suggesting that under stress conditions the parasite still strives for host attachment in an “infect-or-perish” attempt, without being affected by common stress responsive mechanisms. Similarly, the early growth of *C. campestris* and *C. chinensis* under salinity was subordinated to retained growth in length rather than weight accumulation as seen from mass-to-length ratios (Figure 2c) as compared to the nonparasitic species and successful host infection ensured further survival, although at reduced growth rate (Figure 6). Similarly, when dormancy was broken for any reason under unfavorable conditions, the seeds seemed to mobilize any possible resources (Figure 4 and Figure 5) to ensure the early survival.

It should be noted, however, that a single *Cuscuta* spp. plant produces several thousands of seeds [39], which are heterogenic, germinate unevenly and over several years [33]. A rough calculation from the current experiment would lead to the idea that under 200 mM NaCl approximately 15% of all *Cuscuta campestris* seeds would give successfully developing plants—50% attached to host from 30% germinated (Figure 1), also with a significant delay in time. In a saline environment, however, the relatively lower biodiversity suggests fewer and more slowly growing hosts [2], which in turns leads to the understanding that under such conditions the slower germination and growth rate along with fewer survived *C. campestris* would be in conformity to the host availability and would reduce the intraspecific competition within the parasitic plant population. Even though it is probably not an evolutionary adaptation on purpose, the inhibiting effect of salinity on the parasitic plant may be beneficial in the above discussed situation.

## 4. Materials and Methods

### 4.1. Plant Material

*Cuscuta campestris* seeds were collected in August, 2018 in the Thracian Plane, LH 70, 42°27′13″ N 25°30′06″ E. The species was morphologically determined. *Cuscuta chinensis* and *C. japonica* seeds were provided from the collection of Zhejiang Provincial Key Laboratory of Plant Evolutionary Ecology and Conservation, Taizhou University, Taizhou, Zhejiang Province, China. Commercially available *Ipomoea tricolor* seeds were purchased from Sortovi Semena AD, Sofia, Bulgaria and grown to mature plants in greenhouse, from which the seeds for the experiments were collected. *Convolvulus arvensis* and *Calystegia sepium* seeds were collected in the area of Negovan (42°46′08.1″ N 23°24′01.1″ E), Bulgaria and morphologically identified. The average seed weight (per 100 seeds) was as follow: 0.068 ± 0.016 g for *C. campestris*, 0.057 ± 0.005 g for *C. chinensis*, 0.928 ± 0.159 g for *C. japonica*, 3.364 ± 0.636 g for I. tricolor, 1.028 ± 0.107 g for *C. arvensis* and 2.384 ± 0.574 g for *C. sepium* (n = 5).

### 4.2. Experimental Design

Seeds from all species were surface sterilized for 3 min in 70% ethanol, 5 min in 10% commercial bleach, washed in distilled water 5 times, chemically scarified in concentrated sulfuric acid for 15 min to break dormancy and placed on 5 layers of filter paper, soaked with either 0 (control), 100 or 200 mM NaCl in distilled water in 110 mm diameter glass petri dishes. Sodium chloride concentrations were chosen on the basis of preliminary experiments on *C. campestris* alone from a concentration range from 0 to 400 mM NaCl where less than 100 mM NaCl had little or no effect and more than 200 mM NaCl inhibited further seedling growth, although not inhibiting germination completely. Germination was performed in a phytostatic chamber at 27 °C, 16 h light/8 h dark photoperiod. Germination rate was recorded for 10 days. Germination percentage was calculated as following:

(1) $$Germination\;percentage=\;\frac{Number\;of\;germinated\;seeds}{Total\;number\;of\;seeds}\;\times \;100$$

Initial seedling growth was measured as length in cm and fresh mass in mg 7 days after germination of each individual seedling. Further development was assessed as growth in length (cm) and fresh weight (mg) per day after transfer of the seedlings in soil:vermiculite mixture (3:1) in individual pots (nonparasitic species) or on pregrown *Arabidopsis thaliana* plants to serve as hosts (*Cuscuta* spp.) 10 days after transfer (nonparasitic species) or after formation of a secondary stem (*Cuscuta* spp.). Plants were grown in greenhouse conditions at 22 °C, 70% relative humidity and 16 h light/8 h dark photoperiod.

### 4.3. Protein Isolation and Zymographic Analysis

Water-soluble proteins were isolated after the grinding of plant material in liquid nitrogen, and resuspension in 0.1 mL per 0.01 g tissue of phosphate buffered saline (pH 7.2) in absence of protease inhibitors and centrifugation at 10 000 g, 4 °C for 15 min. The protein concentration in the supernatant was measured by Pierce^TM^ BCA Protein Assay Kit (ThermoFisher Scientific, Waltham, MA, USA) and for further experiments all samples were separated by denaturing polyacrylamide gel electrophoresis (SDS PAGE) at 15 µg quantity. Semidenaturing SDS PAGE was performed essentially according to the Laemmli protocol [42] at 12.5% T running gels with copolymerized substrate and 4% T stacking gels, ran on a omniPAGE Mini vertical protein electrophoresis system (Cleaver Scientific, Rugby, UK) at 180 V constant current, followed by 30 min wash in 10% Triton X100, extensive wash in distilled water and overnight incubation at 37 °C in PBS pH 7.2, supplemented with 1M CaCl_2_ [43]. For protease activity the in-gel substrate was 0.1% gelatin and staining was with 0.1% Coomassie Brilliant Blue R-250 in 40% methanol, 10% acetic acid. For α-amylase activity in-gel substrate was 0.2% soluble starch and staining was with iodine solution (10 mM I_2_ and 14 mM KI).

### 4.4. Software and Statistical Analyses

Germination was performed in triplicates, each one consisting of 100 seeds (20 seeds per petri dish for *Cuscuta* spp. and 5 seeds per petri dish for the nonparasitic species). All other measurements were performed on 6 individual seedlings, originating from at least 3 individual petri dishes. Results for parameters of growth are presented as percentage of control to allow comparison between the parasitic and nonparasitic species due to the much bigger seeds and seedlings of nonparasitic species. All data were statistically analyzed using analysis of variance (ANOVA) with level of significance at either *p* ≤ 0.05 or *p* ≤ 0.01 with Tukey’s post hoc test and Levene’s test for homogeneity on JASP 0.9.0.1 (University of Amsterdam, Amsterdam, The Netherlands). For germination percentage the effect of salinity on germination within individual species was tested at the 5th and 10th day. Factorial ANOVA was performed to test the effect of species, salinity and interaction between them on the final germination. Final germination percentage (10th day) of the controls of each species was assumed as 100% and germination percentage under salinity was recalculated:

(2) $$Germination\;percentage\;=\;\frac{\begin{array}{c} Number\;of\;\\ germinated\;seeds\; \end{array}}{\begin{array}{c} Number\;of\;germinated\\ seeds\;at\;0\;mM\;NaCl \end{array}}\;\times \;100$$

All values were transformed to angular values (arcsin $\sqrt{\%}$), tested for homogeneity and subjected to two-way ANOVA [44]. All graphs were plotted using GraphPad Prism 8.

## 5. Conclusions

The presented results strongly suggest high sensitivity to salinity of the parasitic plant *Cuscuta campestris* during the early germination events and seedling growth. Although it is a highly invasive and noxious weed with broad ecological plasticity, it showed poorer tolerance to salinity, compared to its nonparasitic relative. In contrast, it is a much more effective parasite under salinity in comparison to the other two *Cuscuta* species. The inability to cope with the negative effects of salt stress may be partially predetermined by the low content of storage compounds in the seeds. Such sensitivity to adverse conditions, however, may be beneficial in an ecological perspective, as lower germination and survival rate under restricted host availability could contribute to better adjustment to the available resources. The germination percentage of the nonparasitic vines was similarly affected, although their further development was less affected than the parasitic species.

## Figures and Tables

**Table 1:** Two-way ANOVA analysis of the effect of species and salinity on the final germination percentage.

Source of Variation	df	Mean Square	F-Value
Species	5	673.8	2.35
Salinity	2	9792.5	34.1 **
Species × salinity	10	418.1	1.46

** *p* ≤ 0.01.

## Data Availability

Not applicable.

## References

[1] Munns R., James R.A., Läuchli A. (2006). Approaches to increasing the salt tolerance of wheat and other cereals. J. Exp. Bot..

[2] Flowers T.J., Colmer T.D. (2008). Salinity tolerance in halophytes. New Phytol..

[3] Flowers T.J. (2004). Improving crop salt tolerance. J. Exp. Bot..

[4] Vallejo A.J., Yanovsky M.J., Botto J.F. (2010). Germination variation in *Arabidopsis thaliana* accessions under moderate osmotic and salt stresses. Ann. Bot..

[5] Gul B., Ansari R., Flowers T.J., Khan M.A. (2013). Germination strategies of halophyte seeds under salinity. Environ. Exp. Bot..

[6] Muscolo A., Sidari M., Anastasi U., Santonoceto C., Maggio A. (2013). Effect of PEG-induced drought stress on seed germination of four lentil genotypes. J. Plant Interact..

[7] Yao S., Lan H., Zhang F. (2010). Variation of seed heteromorphism in Chenopodium album and the effect of salinity stress on the descendants. Ann. Bot..

[8] Jisha K.C., Vijayakumari K., Puthur J.T. (2013). Seed priming for abiotic stress tolerance: An overview. Acta Physiol. Plant..

[9] Kim S.-G., Lee A.-K., Yoon H.-K., Park C.-M. (2008). A membrane-bound NAC transcription factor NTL8 regulates gibberellic acid-mediated salt signaling in Arabidopsis seed germination. Plant J..

[10] Li W., Yamaguchi S., Khan M.A., Weiqiang L., Liu X., Tran L.-S.P. (2016). Roles of Gibberellins and Abscisic Acid in Regulating Germination of Suaeda salsa Dimorphic Seeds Under Salt Stress. Front. Plant Sci..

[11] Lin Y., Yang L., Paul M., Zu Y., Tang Z. (2013). Ethylene promotes germination of Arabidopsis seed under salinity by decreasing reactive oxygen species: Evidence for the involvement of nitric oxide simulated by sodium nitroprusside. Plant Physiol. Biochem..

[12] Munns R., Tester M. (2008). Mechanisms of salinity tolerance. Annu. Rev. Plant Biol..

[13] Møller I.S., Gilliham M., Jha D., Mayo G.M., Roy S.J., Coates J.C., Haseloff J., Tester M. (2009). Shoot Na+ Exclusion and Increased Salinity Tolerance Engineered by Cell Type–Specific Alteration of Na+ Transport in Arabidopsis. Plant Cell.

[14] Chen Z., Cuin T.A., Zhou M., Twomey A., Naidu B.P., Shabala S. (2007). Compatible solute accumulation and stress-mitigating effects in barley genotypes contrasting in their salt tolerance. J. Exp. Bot..

[15] Bose J., Rodrigo-Moreno A., Shabala S. (2014). ROS homeostasis in halophytes in the context of salinity stress tolerance. J. Exp. Bot..

[16] Vera-Gargallo B., Chowdhury T.R., Brown J., Fansler S.J., Durán-Viseras A., Sánchez-Porro C., Bailey V.L., Jansson J.K., Ventosa A. (2019). Spatial distribution of prokaryotic communities in hypersaline soils. Sci. Rep..

[17] Moreno J., Terrones A., Juan A., Alonso M. (2018). Ángeles Halophytic plant community patterns in Mediterranean saltmarshes: Shedding light on the connection between abiotic factors and the distribution of halophytes. Plant Soil.

[18] Latef A.A.H.A., Hashem A., Rasool S., Abd_Allah E.F., Alqarawi A.A., Egamberdieva D., Jan S., Anjum N.A., Ahmad P. (2016). Arbuscular mycorrhizal symbiosis and abiotic stress in plants: A review. J. Plant Biol..

[19] Asano T., Hayashi N., Kobayashi M., Aoki N., Miyao A., Mitsuhara I., Ichikawa H., Komatsu S., Hirochika H., Kikuchi S. (2011). A rice calcium-dependent protein kinase OsCPK12 oppositely modulates salt-stress tolerance and blast disease resistance. Plant J..

[20] Bostock R.M. (2005). Signal Crosstalk and Induced Resistance: Straddling the Line Between Cost and Benefit. Annu. Rev. Phytopathol..

[21] Veste M., Todt H., Breckle S.-W. (2015). Influence of halophytic hosts on their parasites—the case of *Plicosepalus acaciae*. AoB Plants.

[22] Callaway R.M., Pennings S.C. (1998). Impact of a parasitic plant on the zonation of two salt marsh perennials. Oecologia.

[23] Grewell B.J. (2008). Parasite facilitates plant species coexistence in a coastal wetland. Ecology.

[24] Zagorchev L., Albanova I., Tosheva A., Li J., Teofanova D. (2018). Salinity effect on Cuscuta campestris Yunck. Parasitism on *Arabidopsis thaliana* L.. Plant Physiol. Biochem..

[25] Cochavi A., Ephrath J., Eizenberg H., Rachmilevitch S. (2018). *Phelipanche aegyptiaca parasitism* impairs salinity tolerance in young leaves of tomato. Physiol. Plant..

[26] Dawson J.H., Musselman L.J., Wolswinkel P., Dorr I. (1994). Biology and control of Cuscuta. Rev. Weed Sci..

[27] Parker C. (2012). Parasitic Weeds: A World Challenge. Weed Sci..

[28] Costea M., Spence I., Stefanović S. (2011). Systematics of Cuscuta chinensis species complex (subgenus Grammica, Convolvulaceae): Evidence for long-distance dispersal and one new species. Org. Divers. Evol..

[29] Hrusa G.F., Kelch D., Kodira U.C. (2006). Giant dodders 2004–2006. Plant Pest Diagnostics Center.

[30] Pfirter H.A., Ammon H.-U., Guntli D., Greaves M.P., Defago G. (1997). Towards the management of field bindweed (Convolvulus arvensis) and hedge bindweed (*Calystegia sepium*) with fungal pathogens and cover crops. Integr. Pest Manag. Rev..

[31] Sherman T.D., Bowling A.J., Barger T.W., Vaughn K.C. (2008). The Vestigial Root of Dodder (*Cuscuta pentagona*) Seedlings. Int. J. Plant Sci..

[32] Shen S., Xu G., Clements D.R., Jin G., Liu S., Yang Y., Chen A., Zhang F., Kato-Noguchi H. (2016). Suppression of reproductive characteristics of the invasive plant Mikania micrantha by sweet potato competition. BMC Ecol..

[33] Jayasuriya K.M.G.G., Baskin J.M., Geneve R.L., Baskin C.C., Chien C.-T. (2008). Physical Dormancy in Seeds of the Holoparasitic Angiosperm *Cuscuta australis* (Convolvulaceae, Cuscuteae): Dormancy-breaking Requirements, Anatomy of the Water Gap and Sensitivity Cycling. Ann. Bot..

[34] McNeal J.R., Arumugunathan K., Kuehl J.V., Boore J.L., Depamphilis C.W. (2007). Systematics and plastid genome evolution of the cryptically photosynthetic parasitic plant genus Cuscuta(Convolvulaceae). BMC Biol..

[35] Van Der Kooij T.A.W., Krause K., Dörr I., Krupinska K. (2000). Molecular, functional and ultrastructural characterisation of plastids from six species of the parasitic flowering plant genus Cuscuta. Planta.

[36] Vogel A., Schwacke R., Denton A.K., Usadel B., Hollmann J., Fischer K., Bolger A., Schmidt M.H.-W., Bolger M.E., Gundlach H. (2018). Footprints of parasitism in the genome of the parasitic flowering plant Cuscuta campestris. Nat. Commun..

[37] Yoneyama K., Awad A.A., Xie X., Takeuchi Y. (2010). Strigolactones as Germination Stimulants for Root Parasitic Plants. Plant Cell Physiol..

[38] Charles D.J., Singh M., Sanwal G.G. (1982). Biochemical changes during germination and seedling growth in *Cuscuta campestris*. Physiol. Plant..

[39] Tănase M., Stanciu M., Moise C., Gheorghe M. (2012). Ecological and economic impact of dodder species (*Cuscuta* spp. Convolvulaceae) on pratological ecosystems. J. Hortic. For. Biotechnol..

[40] Yergin-Ozkan R., Tepe I. (2018). Emergence characteristics and germination physiology of smoothseed alfalfa dodder (*Cuscuta approximata* Bab.). Fresenius Environ. Bull..

[41] Li J., Hettenhausen C., Sun G., Zhuang H., Li J.-H., Wu J. (2015). The Parasitic Plant Cuscuta australis Is Highly Insensitive to Abscisic Acid-Induced Suppression of Hypocotyl Elongation and Seed Germination. PLoS ONE.

[42] Laemmli U.K. (1970). Cleavage of structural proteins during the assembly of the head of bacteriophage T4. Nature.

[43] Manchenko G.P. (2002). Handbook of Detection of Enzymes on Electrophoretic Gels.

[44] Raccuia S.A., Cavallaro V., Melilli M.G. (2004). Intraspecific variability in *Cynara cardunculus* L. var. sylvestris Lam. *Sicilian populations*: Seed germination under salt and moisture stresses. J. Arid. Environ..

